# Uranyl Sulfate Nanotubules Templated by *N*-phenylglycine

**DOI:** 10.3390/nano8040216

**Published:** 2018-04-03

**Authors:** Oleg I. Siidra, Evgeny V. Nazarchuk, Dmitry O. Charkin, Nikita V. Chukanov, Wulf Depmeier, Sergey N. Bocharov, Mikhail I. Sharikov

**Affiliations:** 1 Department of Crystallography, Saint-Petersburg State University, University emb. 7/9, 199034 St. Petersburg, Russia; e_nazarchuk@mail.ru (E.V.N.); bocharovsergei@mail.ru (S.N.B.); 2 Nanomaterials Research Center, Kola Science Center, Russian Academy of Sciences, Apatity, 184200 Murmansk Region, Russia; 3 Department of Chemistry, Moscow State University, Vorobievy Gory 1, 119991 Moscow, Russia; charkin@inorg.chem.msu.ru (D.O.C.); mikhail.sharikov.92@mail.ru (M.I.S.); 4 Institute of Problems of Chemical Physics, Chernogolovka, 142432 Moscow Region, Russia; chukanov@icp.ac.ru; 5 Institut für Geowissenschaften der Universität Kiel, Olshausenstr 40, D-24098 Kiel, Germany; wulf.depmeier@ifg.uni-kiel.de

**Keywords:** nanostructured materials, nanotubes, actinide materials, uranium, sulfates, microporous compounds, organically templated compounds

## Abstract

The synthesis, structure, and infrared spectroscopy properties of the new organically templated uranyl sulfate Na(phgH^+^)_7_[(UO_2_)_6_(SO_4_)_10_](H_2_O)_3.5_ (**1**), obtained at room temperature by evaporation from aqueous solution, are reported. Its structure contains unique uranyl sulfate [(UO_2_)_6_(SO_4_)_10_]^8−^ nanotubules templated by protonated *N*-phenylglycine (C_6_H_5_NH_2_CH_2_COOH)^+^. Their internal diameter is 1.4 nm. Each of the nanotubules is built from uranyl sulfate rings sharing common SO_4_ tetrahedra. The template plays an important role in the formation of the complex structure of **1**. The aromatic rings are stacked parallel to each other due to the effect of π–π interaction with their side chains extending into the gaps between the nanotubules.

## 1. Introduction

From the point of view of environmental chemistry and mineralogy, uranyl sulfates are of great interest as possible alteration products of nuclear waste [1,2,3]. Uranyl oxysalt structures often contain large open spaces like channels or cavities and are therefore materials with potential practical importance, e.g., for sorption, separation, and catalysis processes in the actinide industry, or for the management of spent nuclear fuel [4]. The crystal chemistry of U(VI) compounds containing various tetrahedral oxo-anions (e.g., SO_4_, SeO_4_, VO_4_, PO_4_ etc.) is dominated by two-dimensional (2*D*) structural motifs, resulting from the well-known and strong directional anisotropy of the bond distribution in the uranyl coordination geometry [5]. In contrast, framework structures are less common for uranyl oxysalts [6], and the current knowledge concerning purely inorganic nanotubules is limited to three uranyl selenate based species [7,8,9], which differ in their inner diameters, viz., two of them are 0.7 nm [7,8] and the other one is 1.5 nm [9]. In other, not purely inorganic, U(VI) compounds, such as metal–organic or hybrid systems, the number of structures containing nanotubules is higher [10,11,12]. 

Herein, we report on the synthesis, structure, and properties of a new organically templated uranyl sulfate of formula Na(phgH^+^)_7_[(UO_2_)_6_(SO_4_)_10_](H_2_O)_3.5_ (**1**), where phgH^+^ is protonated *N*-phenylglycine, featuring unique uranyl sulfate nanotubules, which was obtained at room temperature by evaporation from aqueous solution.

## 2. Materials and Methods 

### 2.1. Synthesis

**Caution!** Uranium acetate UO_2_(CH_3_COO)_2_ used in this study contains depleted uranium. Standard precautions for handling radioactive and toxic substances should be followed.

**Figure 1:**
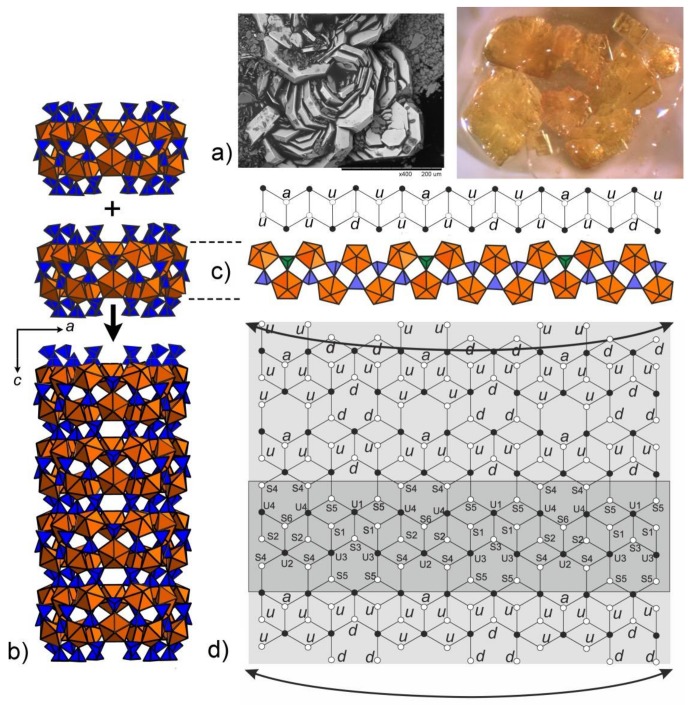
SEM image (Field of view is 0.4 mm) and image under an optical microscope (Field of view is 5 mm) of the platy crystal clusters of **1** (**a**). Polyhedral representation of the uranyl sulfate nanotubule in the structure of **1** (**b**). Uranyl and sulfur coordination polyhedra are shown in orange and blue respectively. The nanotubule in **1** consists of uranyl sulfate rings sharing common SO_4_ tetrahedra. Notionally, the rings can be unfolded into single chains previously known from a number of uranyl oxysalts with tetrahedral anions. An unfolded version of the ring and the corresponding graph are shown. The *u* and *d* symbols identify ‘up’ and ‘down’ orientations of the S–O_t_ bonds in SO_4_. UO_7_ bipyramids and SO_4_ tetrahedra are symbolized by black and white vertices, respectively. The *a* symbol designates tetrahedra (shown by green polyhedra) with orientational disorder (**c**). The topology of the layer corresponding to the unfolded uranyl-sulfate nanotubule in the structure of **1** is shown in (**d**).

*N*-phenylglycine (phg) was prepared as follows [13]: chloroacetic acid (10 mmol) was neutralized by sodium carbonate. To the solution of sodium chloroacetate, 10 mmol of freshly distilled aniline was added slowly and refluxed until complete reaction of aniline. Upon cooling, a precipitate was formed which was separated and washed with cold water. The ^1^H NMR spectra of the yellowish raw product indicated the presence of two compounds: the target phg and a second one identified as a cyclic anilide of *N*,*N*-aniline diacetic acid (Figure S1). The phg could be separated by extracting the admixture into diethyl ether (wherein phg is insoluble). In a test experiment, 0.1 mmol of UO_2_(CH_3_COO)_2_ and 0.05 mmol of Na_2_CO_3_ were dissolved in 5 mL of 0.1M H_2_SO_4_; 0.0151 g of the raw product was then added to form a clear yellowish solution which was poured onto a watch glass and left to evaporate. After two days, the solution turned tarry and dark colored; clusters of small yellowish platy crystals (35% yield) (Figure 1a) were formed within two more days. Surprisingly, the use of pure phg yielded only yellow glassy films upon drying. Attempts to increase the content of H_2_SO_4_, or to replace it with H_2_SeO_4_ in stoichiometric amounts yielded the formation of black tarry product with no crystals formed. The synthesis was repeated with the addition of *N*,*N*-aniline diacetic acid to the pure phg and turned out to be successful again, as in the test experiment. Though the role of the side organic product (cyclic anilide of *N*,*N*-aniline diacetic acid) remains unclear, we suggest that its oxidation/decomposition products provide the gel-like growth medium for the crystals of **1**. Qualitative electron microprobe analysis TM3000 (Hitachi, Tokyo, Japan) revealed no other elements, except U, S, and Na, with an atomic number greater than 11 (Na).

### 2.2. Single Crystal X-ray Diffraction Studies

Single crystals of **1** were mounted on thin glass fibers for X-ray diffraction (XRD) analysis and tested using a X8 APEX II X-ray diffractometer (Bruker, Karlsruhe, Germany) with a fine-focus X-ray tube delivering Mo*Kα* radiation, *λ* = 0.71073 Å at 50 kV and 40 mA. More than a hemisphere of three-dimensional data was collected with a frame width of 0.5° in ω, and 30 s exposition time per frame. The diffraction data were integrated and corrected for absorption using a multi-scan type model integrated in the APEX2 and SADABS programs (Bruker, Madison, WI, USA). The unit cell parameters of **1** (*a* = 44.001(10) Å, *c* = 10.367(2) Å, *V* = 17382(9) Å^3^) were determined and refined by least-squares techniques on the basis of 37,916 reflections. The value of the|E^2^-1|parameter, 0.738, indicated a high probability of a non-centrosymmetric space group, which was confirmed by the subsequent structure solution and refinement. The refined Flack parameter (*x* = 0.180(9)) indicated that the studied crystal of **1** has almost pure absolute configuration. The structure was solved in space group *R*3*m* by direct methods and refined to *R*_1_ = 0.028 (*wR*_2_ = 0.076) for 8212 reflections with |*F*_o_| ≥ 4*σF* by using the SHELXL–2013 program implemented in the WinGX program package (University of Glasgow, Glasgow, Great Britain). The final model included coordinates and anisotropic displacement parameters for all atoms, except hydrogen atoms which could not be localized. Data collection refinement parameters and detailed crystallographic information are provided in Table 1. CCDC 1579352 contains the supplementary crystallographic information for **1**.

### 2.3. High-Temperature Powder X-ray Diffraction Studies

**Figure 2:**
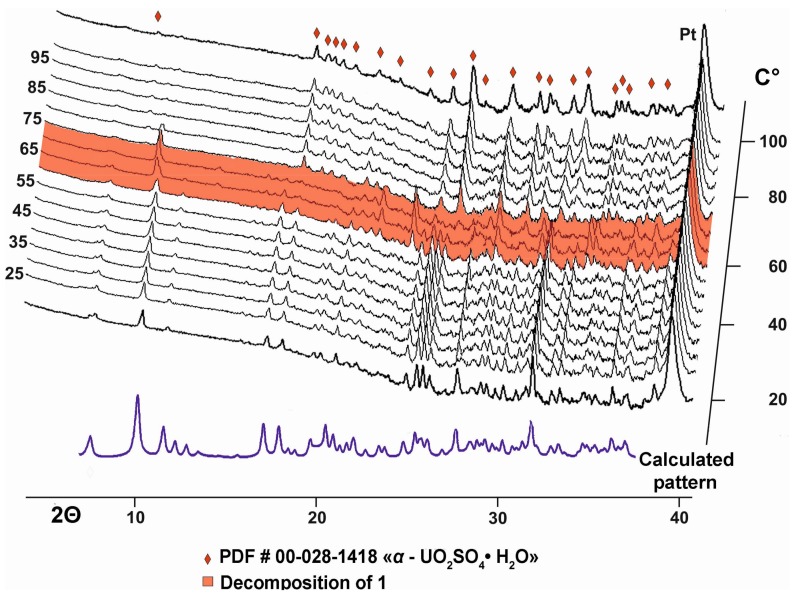
Evolution of the powder diffraction patterns of **1** as a function of temperature. Patterns in the temperature range of decomposition of **1** into “UO_2_SO_4_·H_2_O” (PDF # 00-028-1418) are marked in red.

Some of the obtained single crystals were ground using an agate mortar and then subjected to a high-temperature powder X-ray diffraction analysis in air by means of a Ultima X-ray diffractometer (Cu*Kα* radiation) (Rigaku, Tokyo, Japan) equipped with a high-temperature camera HTA 1600 (Rigaku, Tokyo, Japan). The samples were prepared from heptane suspension on Pt-Rh plates. Temperature steps were 5 K in the range 20–110 °C. The evolution of the powder diffraction patterns with increasing temperature is shown in Figure 2.

### 2.4. Infrared Spectroscopy

In order to obtain infrared (IR) absorption spectra, a powdered sample of **1** was mixed with dried KBr, pelletized, and the spectra recorded using an ALPHA FTIR spectrometer (Bruker Optik GmbH, Ettlingen, Germany) with a resolution of 4 cm^−1^. In total, 16 scans were accumulated. The IR spectrum of an analogously prepared pellet of pure KBr was used as a reference. 

### 2.5. Crystal Surface Microtopography

To determine a possible correlation of the presence of nanotubules in the structure with the micromorphology of the crystal faces, the surface of the crystals of **1** was studied by means of an atomic force microscopy (AFM) (NT-MDT, Ntegra Prima, Zelenograd, Russia) in a contact mode with the measurement of the cantilever (ETALON HA-C, NT-MDT, Zelenograd, Russia) DFL signal. Crystals were studied in air and without additional surface treatment. Results are reported in the caption of Figure S2.

## 3. Results and Discussion

Compound **1** is stable up to approximately 60 °C when the diffraction maxima gradually disappear (Figure 2). At 75 °C, peaks of “UO_2_SO_4_·H_2_O” (PDF # 00-028-1418) with unknown structure appear indicating a loss of organic molecules, whereas Na is probably accumulating in an amorphous phase.

The structure of **1** contains four symmetrically independent U(VI) and six S(VI) atoms. Each uranium atom is strongly bonded to two O atoms to form a uranyl ion, UO_2_^2+^ (U1-O20 = 1.745(12) Å, U1-O7 = 1.773(13) Å; U2-O6 = 1.773(11) Å, U2-O21 = 1.780(12) Å; U3-O12 = 1.753(8) Å, U3-O15 = 1.764(8) Å; U4-O14 = 1.749(8) Å, U4-O19 = 1.758(8) Å). Each of the uranyl ions is further coordinated by five O atoms at the equatorial vertices of UO_7_ pentagonal bipyramids (<U1-O_eq_> = 2.388 Å, <U2-O_eq_> = 2.379 Å, <U3-O_eq_> = 2.392 Å, <U4-O_eq_> = 2.402 Å,). S^6+^ cations are tetrahedrally coordinated. One of the S sites is orientationally disordered with the S6 site split into the S6A and S6B subsites. The average S–O bond-lengths in the ordered SO_4_ tetrahedra, 1.46–1.48 Å, are consistent with the average value of 1.475 Å given for sulfates in general [14]. UO_7_ bipyramids share common corners with S1O_4_, S2O_4_, S3O_4_ and S6O_4_ tetrahedra, thus forming the rings shown in Figure 1b. 

**Figure 3:**
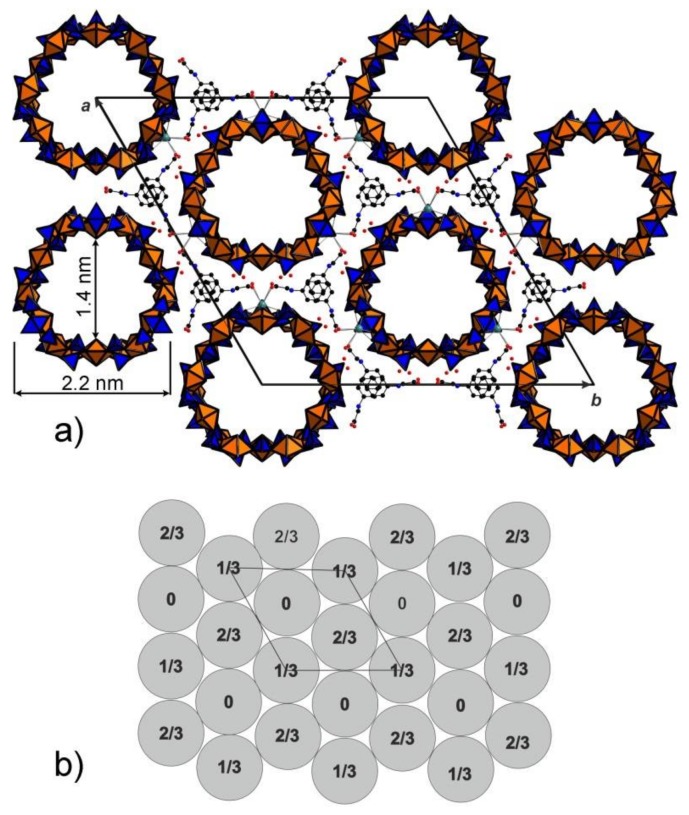
General view of the structure of **1** along the *c* axis showing the cross-section of the nanotubules (**a**). Phenylglycine molecules between the tubules are parallel to each other due to the effect of π–π interactions. Scheme of arrangements of nanotubules in the structure of **1** (**b**). The action of space group *R*3*m* results in neighbouring nanotubules being shifted by 1/3 or 2/3 along the *c* axis in accordance with the *R*-centering.

**Figure 4:**
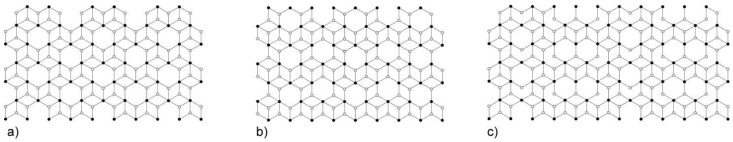
Nodal representation of [(UO_2_)_3_(*T*O_4_)_5_]^4−^ (*T* = Mo, Se) layers in [C_3_N_2_H_12_](H_3_O)_2_[(UO_2_)_3_(MoO_4_)_5_] [16], Na_6_[(Np^5+^O_2_)_2_(Np^6+^O_2_)(MoO_4_)_5_](H_2_O)_13_ [17], Mg_2_[(UO_2_)_3_(SeO_4_)_5_](H_2_O)_16_ [18], *M*_2_[(UO_2_)_3_(SeO_4_)_5_](H_2_O)_16_ (*M* = Co., Zn) [19] (**a**). The topology of the layer corresponding to the unfolded uranyl-selenate nanotubules in the structures of K_5_[(UO_2_)_3_(SeO_4_)_5_](NO_3_)(H_2_O)_3.5_ [7], (H_3_O)_2_K[(H_3_O)@([18]crown-6)][(UO_2_)_3_(SeO_4_)_5_](H_2_O)_4_ [8] (**b**) and. (C_4_H_12_N)_14_[(UO_2_)_10_(SeO_4_)_17_(H_2_O)] [9] (**c**).

The rings can be unfolded into single chains which were previously reported in the structures of different uranyl oxysalts with tetrahedral anions [15] (Figure 1c). Additional S4O_4_ and S5O_4_ tetrahedra provide the stacking of the rings into [(UO_2_)_6_(SO_4_)_10_]^8−^ tubules extending parallel to the *c* axis (Figure 3a) and packed in a hexagonal-type fashion (Figure 3b). We were unable to determine the positions of any C, N, or O atoms inside the tubes, obviously due to their heavily disordered arrangement. Various attempts using the algorithms and programs implemented in the Platon software package remained unsuccessful. The internal diameter of the tubules measured as the distance between the closest oxygen atoms across the tubule is 13.5 Å, which is close to the inner diameter of uranyl selenate nanotubules in (C_4_H_12_N)_14_[(UO_2_)_10_(SeO_4_)_17_(H_2_O)] [9]. It is worth noticing that for this compound, it was likewise impossible to localize the organic molecules inside the tubules. In Reference [9], the [(UO_2_)_10_(SeO_4_)_17_(H_2_O)]^14−^ uranyl selenate nanotubules contain water molecules coordinating the (UO_2_)^2+^, whereas the uranyl cations in the [(UO_2_)_6_(SO_4_)_10_]^8−^ nanotubules of **1** do not. The structural topology of the [(UO_2_)_6_(SO_4_)_10_]^8−^ tubule can be revealed by the nodal representation of its unfolded version (Figure 1d). Here, U and S polyhedra are symbolized by black and white nodes, respectively. Two nodes are linked by an edge if the respective polyhedra share a common corner. It can be seen that the topology of [(UO_2_)_6_(SO_4_)_10_]^8−^ *tubules* in **1** is similar to that of the [(UO_2_)_3_(*T*O_4_)_5_]^4−^ (*T* = Cr, Se) *layers* (Figure 4a) previously reported for several uranyl and neptunyl molybdates and uranyl selenates, viz., [C_3_N_2_H_12_](H_3_O)_2_[(UO_2_)_3_(MoO_4_)_5_] [16], Na_6_[(Np^5+^O_2_)_2_(Np^6+^O_2_)(MoO_4_)_5_](H_2_O)_13_ [17], Mg_2_[(UO_2_)_3_(SeO_4_)_5_](H_2_O)_16_ [18], *M*_2_[(UO_2_)_3_(SeO_4_)_5_](H_2_O)_16_ (*M* = Co., Zn) [19]. However, the “… up-down …” orientations of the *T*-O*_t_* (*T* = Mo, Se) bonds relative to the plane of the layers differ from those with *T* = S as observed in **1**. The topology (Figure 4b,c) of the unfolded uranyl-selenate nanotubules previously reported in References [7,8,9] is very different from that of **1**. 

The protonated phg molecules stack in ordered piles in the gaps between the tubes probably forming NH…O hydrogen bonds with the apical oxygens of the SO_4_ tetrahedra; additional strong links are provided by sodium cations coordinating oxygens from both sulfate and carboxyl groups. The arrangement of the ordered organic part of the structure may thus be considered as columns of phgH^+^ cations, likely exhibiting π–π interactions between their parallel phenyl rings with their (CH_2_COOH) side chains “embracing” the tubes. There is a good size match between the size of this part and the inter-tube space which reflects the templating effect of both Na^+^ and phgH^+^ which can be supposed to not only assist the formation of the nanotubes, but also to determine their size. 

**Figure 5:**
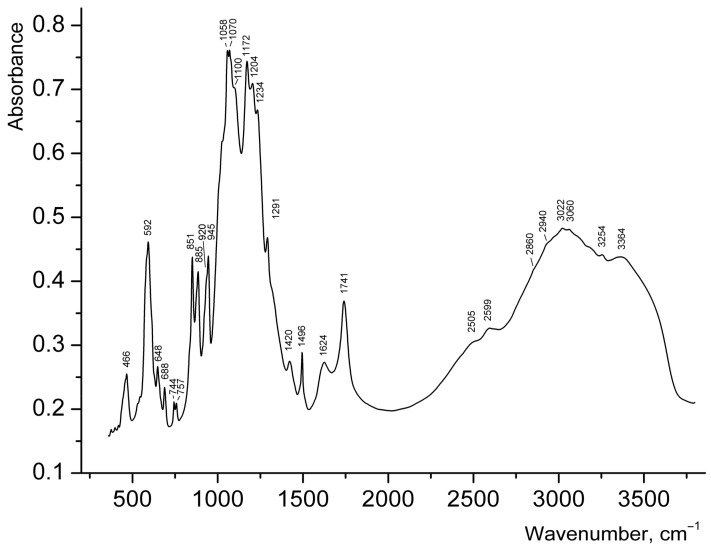
IR spectrum of **1**.

Broad strong bands (Figure 5) in the range of 3200–3500 cm^−1^ in the IR spectrum of **1** can be assigned to N–H and O–H stretching vibrations. Taking into account that salts of secondary amines do not contain strong bands above 3320 cm^−1^ [20,21] the band at 3364 cm^−1^ can be assigned to covalent O–H bonds in H_2_O. Bands of H–O–H bending vibrations in the range of 1700–1800 cm^−1^ are usually indicative of hydronium cations [22,23,24], but this band could not be reliably detected in the IR spectrum of **1**, because of the presence of the strong band of C=O stretching vibrations of carboxyl groups at 1741 cm^−1^. The band at 1624 cm^−1^ may correspond to H–O–H bending vibrations of H_2_O molecules, but it is to be noted that bands in the region from 1620 to 1560 cm^−1^ are also characteristic for secondary amine salts (H–N–H bending vibrations [20]). The broad IR absorption in the range of 2800–3200 cm^−1^ is mainly due to N–H stretching vibrations. The bands at 2599 and 2505 cm^−1^ correspond to acid groups.

Wavenumbers of other absorption bands (cm^−1^) and their assignments are as follows: 1496, 1420 (vibrations of the aromatic ring), 1291 (bending vibrations of *R*_2_NH_2_^+^), 1058–1234 [ν_3_(*F*_2_) asymmetric stretching vibrations of SO_4_^2−^ ions], 851–945 [ν_3_ antisymmetric stretching vibrations of UO_2_^2+^ ions], 592 [ν_4_(*F*_2_) asymmetric bending vibrations of SO_4_^2−^ ions], 466 [ν_2_(*E*) symmetric bending vibrations of SO_4_^2−^ ions]. Weak features in the ranges of 3000–3100 and 2800–3000 cm^−1^, observed on the background of a broad band, correspond to C–H bonds belonging to aromatic and aliphatic hydrocarbon groups respectively. The assignment of relatively weak bands in the range 648–757 cm^−1^ is ambiguous; some of these bands may be tentatively assigned to bending vibrations involving C–H bonds.

According to the correlation *d*_U–O_ (Å) = 81.2ν_3_^−2/3^ + 0.895 for uranyl groups [25], the ν_3_ bands at 945, 920, 885, and 851 cm^−1^ correspond to the mean U–O bond lengths of 1.738, 1.753, 1.776, and, 1.799 Å respectively, which is in good agreement with the values determined from X-ray structural data discussed above. Splitting of the band of asymmetric stretching vibrations of SO_4_^2−^ ions into six components reflects the presence of multiple non-equivalent sulfate groups in the structure of **1**. Weak distortion of SO_4_ tetrahedra is in agreement with the absence of strong bands of symmetric stretching vibrations of SO_4_^2−^ ions: only a shoulder is observed at about 1000 cm^−1^. Taking into account the absence of long S–O distances in the structure of **1**, the bands of acid groups at 2599 and 2505 cm^−1^ cannot be assigned to S–OH groups.

Despite the fact that the content of the inner part of the tubes could not be localized (due to its strong disorder) by means of the single-crystal X-ray analysis, some conclusions can be drawn based on the features of the IR spectrum described above, supplemented by charge balance considerations: (i) Characteristic bands of H_3_O^+^ groups could not be revealed in the spectrum of **1**; (ii) Disordered protonated phg molecules fill the inner part of uranyl sulfate tubules; (iii) Other organic matter which was present in the synthesis has not been incorporated into the structure of 1 as characteristic bands of its molecules are not observed, in particular those expected at ~3440 cm^−1^, 1270–1287, 1027–1029, 867–877, and 820–830 cm^−1^ for aniline [26] and cyclic anilide of *N*,*N*-aniline diacetic acid. 

## 4. Final Remarks

During the last 10 years, numerous attempts at obtaining nanotubular materials in a sulfate-bearing uranyl system have been without success. The present study has now demonstrated that uranyl-based nanotubes can indeed occur in such a system as well and their occurrence is not restricted to selenium containing systems. This suggests that further exploration of uranyl-based systems with other tetrahedral oxoanions is worthwhile despite the obvious predominance of layered structures in these materials. The uranyl nanotubes reported herein can notionally be unfolded into layered prototype structures in a way similar to how carbon nanotubes are related to the graphene layers of graphite [27], or how MoS_2_ nanotubes form from layers in molybdenite [28]. Undiscovered, but conceivable nanotubular structures, based on uranyl polyhedra and other tetrahedral TO_4_ oxoanions, promise to show great structural variety because of the well-known flexibility of the U-O-T links.

The uranyl selenate nanotubules described in References [7,8] can be described as “narrow” as their diameter is only half of that found in **1**. The “wider” nanotubules of [(UO_2_)_10_(SeO_4_)_17_(H_2_O)]^14−^ [9] contain water in their walls. Compared to these two cases, the [(UO_2_)_6_(SO_4_)_10_]^8−^ nanotubules in **1** are unique to date as they occur in a sulfate containing system and their tube walls are unhydrated. The (UO_2_)_6_(SO_4_)_10_]^8−^ tubule in **1** has several isomorphous topologies among layered uranyl selenates and molybdates; under appropriate conditions, the formation of similar nanotubules is conceivable in these systems as well. Supposedly, the organic template with its aromatic ring and the functional side chain is essential for the formation of the structure of **1**, whereby π–π interactions and hydrogen bonds probably play an important role. Unfortunately, the given limitations of the present experiment did not allow for a determination of these fine details. These limitations are mostly due to (i) inherent difficulties of studying light elements in the presence of heavy uranium using X-ray diffraction methods (ii) strong disorder of the organic part and water and (iii) possible defects of the crystals used because of their growth in a complex, little-known medium. 

Recently, various mechanisms for the formation of uranyl selenate nanotubules were suggested [29]. The strong disorder in the interior of the nanotubules in Reference [9] did not allow the establishment of a well-defined model of the “large” nanotubules formation. In References [7,8], it was proposed that the K^+^ ion was responsible for the formation of “small” nanotubules and of curved topologies in selenate systems in general. This seems to be different in **1**, which contains Na and an additional organic template. It could be hypothesized that phenylglycine template forms self-assembled cylindrical micelles in solution as a result of π–π interactions between the phenyl rings, with the subsequent assemblage of uranyl cations and sulfate anions around the template. The sodium cation coordinates both the sulfate anions of the framework and the carboxylic groups. The role of organic admixtures in the crystal growth process is less well understood. We suggest that the tar formed from the cyclic anilide of the *N*,*N*-aniline diacetic acid and the unreacted aniline serves as a gel medium for the nucleation and growth of the crystals. Selenic acid, as a much more powerful oxidizing agent, would destroy both aniline and phenylglycine, thus preventing gel formation and the formation of nanotubules similar to those occurring in the sulfate system. The strong structural control of the nanotubules by the structure of the organic template suggests that any ion-exchange reactions are unlikely to occur. Further syntheses in various chemical systems, and the accumulation of data on uranium nanotubes formation, may help in predicting possible applications of uranyl-based nanotubes, e.g., the utilization of depleted uranium or separation in the actinide industry.

## Figures and Tables

**Table 1:** Crystallographic information and single crystal refinement parameters for Na(phgH^+^)_7_[(UO_2_)_6_(SO_4_)_10_](H_2_O)_3.5_ (**1**).

**Crystal Data**	
Crystal system	trigonal
Space group	*R*3*m* (No. 160)
*a* (Å)	44.001(10)
*c* (Å)	10.367(2)
*V* (Å^3^)	17382(9)
*Z*	1
*ρ*_calc_ (g cm^−3^)	2.543
Crystal size (mm^3^)	0.15 × 0.15 × 0.10
**Data Collection**	
Diffractometer	Bruker X8 APEX II (CCD)
Radiation, λ (Å)	Mo*Kα*, 0.71073
*μ* (mm^−1^)	12.91
*θ* range (°)	1.85–28.00
No. of measured reflections	37916
Total number of reflections (*R*_int_)	8480 (0.04)
Unique reflections with |*F*_o_| ≥ 4*σF*	8212
**Refinement**	
Refinement method	Full-matrix least-squares on F^2^
Weighting coefficients *a*, *b*	0.03680, 271.9710
Data/restraints/parameters	8480/1/455
*R*_1_, *wR*_2_ (|*F*_o_| ≥ 4*σF*)	0.028, 0.076
*R*_1_, *wR*_2_ (all data)	0.029, 0.077
GoF	1.058
largest diff. peak and hole (e Å^−3^)	2.951, −0.987

## Supplementary Materials

The following are available online at http://www.mdpi.com/2079-4991/8/4/216/s1, Figure S1: ^1^H NMR spectra of the initial reagent, Figure S2: Surface microtopography.
