# Supplementary material for: Uranyl Sulfate Nanotubules Templated by N-phenylglycine
Source: Nanomaterials (Basel). 2018 Apr 3;8(4):216. doi: 10.3390/nano8040216 (PMC5923546; doi:10.3390/nano8040216)
Supplement: Supplementary file 1 [file nanomaterials-08-00216-s001.pdf]

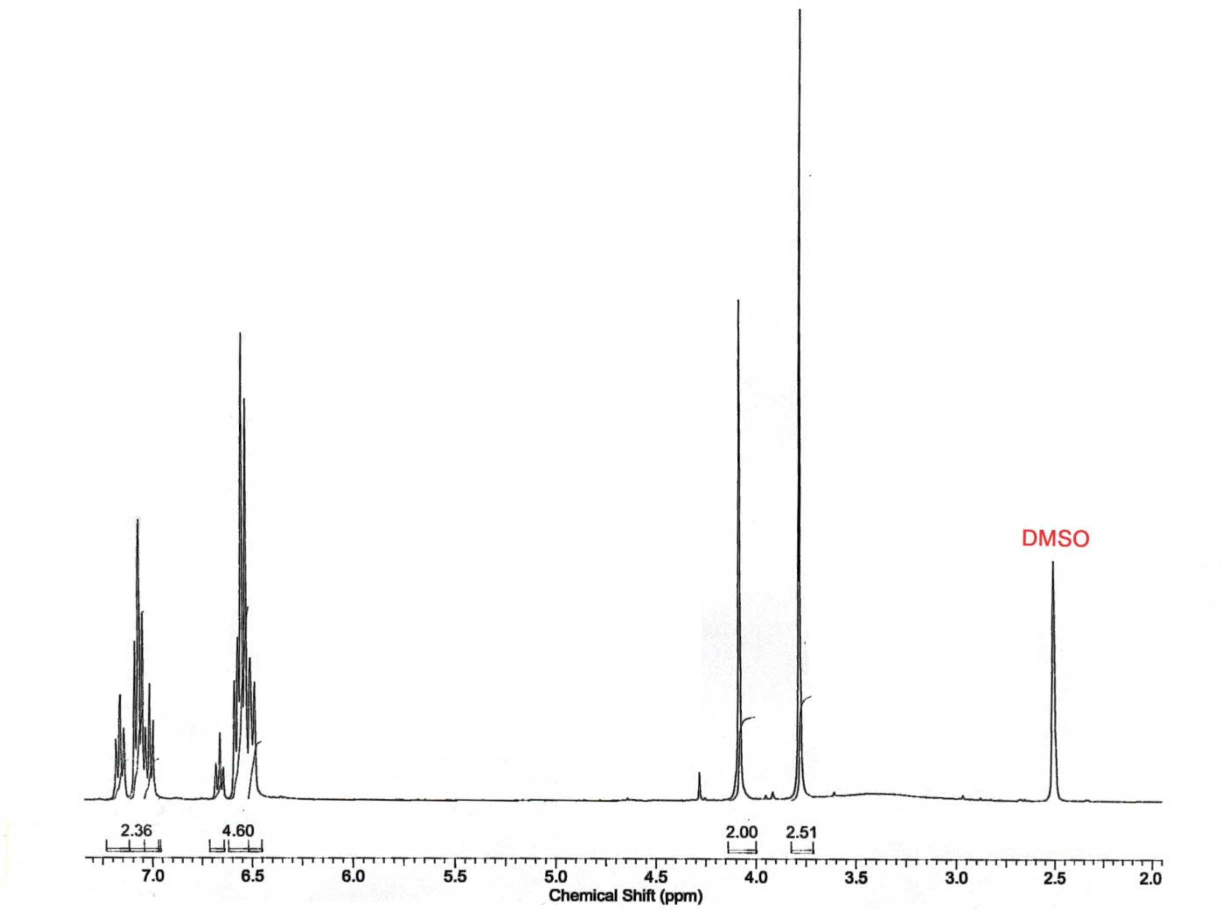

Figure S1. ^{1}H NMR spectra of the initial reagent.

The initial mixture was found to contain *ca.* 90% of the target phenylglycine, and *ca.* 10% of a compound tentatively identified as cyclic anilide of anilinediacetic acid on the basis of NMR spectra. Minute traces of aniline were also detected. It is thus likely that the organic admixture (formed via condensation of aniline diacetic acid with the unreacted aniline) is oxidized by U^{VI} to yield the dark tarry products necessary for the growth of **1**.

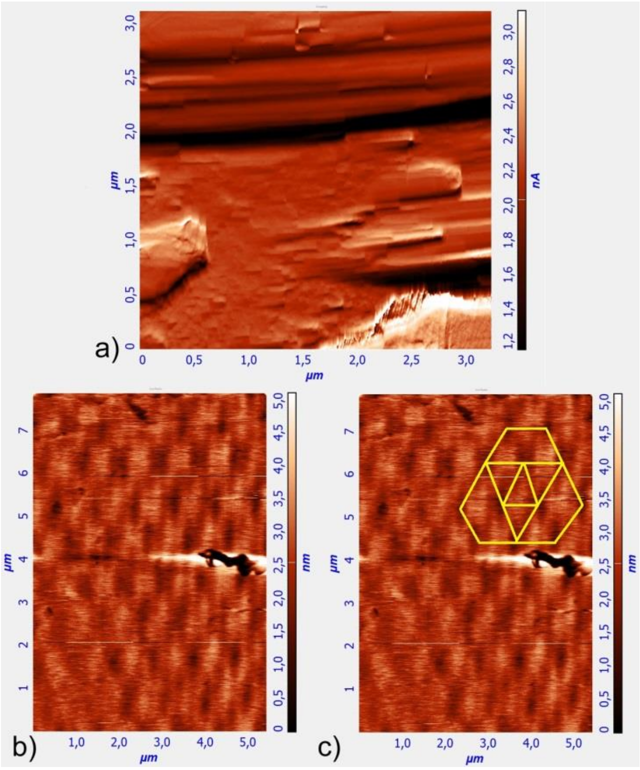

Figure S2. Surface microtopography of the prism faces of **1** showing prismatic needles (a). Ordered structure observed on the pinacoid crystal faces of **1** (b) with highlighted hexagonal packing (c) formed by the ends of the prismatic needles.

The faces of the prism and pinacoid of the crystals of **1** were investigated by AFM. On the prism faces growth forms are represented by the needles (Fig. 2a) up to 900 nm thick, which in turn split into smaller ones, with the thinnest of 15 nm observed. The smaller forms could not be registered due to the large angles of inclination of the studied surfaces. Highly ordered packing with a distance between the elements of 800-850 nm and the height of the elements 1-1.5 nm is observed on some of the faces (Fig. 2b). The hexagonal packing formed by the ends of the prismatic needles is slightly deformed (Fig. 2c) most probably due to the large mutual inclination of the studied crystal face and cantilever. The growth of the crystals of **1** occurs by the condensation of the thin prisms elongated along the *c* axis into larger prismatic forms.
